# Sequence and phylogenetic analysis of the mitochondrial genome for the Poyang Lake, *Misgurnus anguillicaudatus* (natural diploid loach)

**DOI:** 10.1080/23802359.2019.1679675

**Published:** 2020-06-17

**Authors:** Guisheng Zhang, Hongmei Zhang, Guihong Zhao, Xi Yang, Tian Zhang, Qiang Liu, Yan Wang, Guosong Zhang, Daoyu Zhu

**Affiliations:** a School of agriculture and bioengineering, Heze University, Heze, Shandong, China; b E-commerce office, Heze information engineering school, Heze, Shandong, China

**Keywords:** Poyang loach, *Misgurnus*, mitogenome, conservation

## Abstract

*Misgurnus anguillicaudatus* is a highly valued, aquaculture-relevant food fish in East Asian countries. In this study, the complete mitochondrial genome of Poyang loach was obtained by PCR. The genome is 16,646 bp in length, including 2 ribosomal RNA genes. 13 protein-coding genes, 22 transfer RNA genes, and a non-coding control region, the gene composition and order of the species were similar to most reported from other vertebrates. The phylogenetic tree showed that *Misgurnus* family got together for one branch, which includes Poyang *M. anguillicaudatus*, and the other loaches had their own branches.

Cyprinid loach, *Misgurnus anguillicaudatus* (Cypriniformes; Cobitidae), a small-sized freshwater fish species, is a highly valued, aquaculture-relevant food fish in East Asian countries. This loach species has also been given much attention as an important model organism to study developmental biology, polyploidy evolution and genetic breeding (Zhang et al. 2018; Liang et al. 2018a). Because of overfishing and environmental pollution of Poyang Lake, the number of natural wild *M. anguillicaudatus* has been sharply decreased in these years. Therefore it is very important to characterize the complete mitogenome of this species, which could be a fundamental basis to address genetic identity and diversity in future conservation programme of this rarely occurring (Lee et al. 2017).

In this study, we sequenced the complete mitogenome of *M. anguillicaudatus* with a GenBank accession number MN116750. The voucher specimen was collected from Poyang Lake, north latitude 28°45″ and east longitude 116°20″, Jining city, China. They were preserved in 95% alcohol, which was stored in biology herbarium of Heze University (Accession number HZ10050). All DNA was extracted using Phenol-Chloroform extraction method and stored at −80 °C. The mitogenome was amplified by primers, which were initially published (Zeng et al. 2012). The entire mitogenome sequence of Poyang loach was 16,646 bp in length, consisting of 13 protein-coding genes (PCGs), 2 ribosomal RNA (rRNA) genes, 22 transfer RNA (tRNA) genes, 1 replication origin (OL), and 1 control region (D-loop). From the base composition analysis, the percent A + T content was 58.0% (29.77% for A, 28.24% for T, 25.63% for C, and 16.36% for G). Twelve PCGs, 14 tRNA genes and 2 rRNA genes were located on the heavy strand (H-strand), while one PCG (*ND6*) and eight tRNA genes (*tRNA^Gln^*, *tRNA^Ala^*, *tRNA^Asn^*, *tRNA^Cys^*, *tRNA^Tyr^*, *tRNA^Ser^*, *tRNA^Glu^*, and *tRNA^Pro^*) on the light strand (L strand). Eight PCGs (*ND1*, *COI*, *COII*, *ATP8*, *ATP6*, *ND4L*, *ND5*, and *ND6*) were terminated with a TAA stop codon and three PCGs (*ND2*, *ND3*, and *ND4*) ended with TAG. On the other hand, remaining two PCGs (*COIII* and *CYTB*) ended with the incomplete stop codon represented as a single T. Frame overlapping occurred at three pairs of PCGs. *ATP8* and *ATP6* overlapped by 10 nucleotides (nt), *ND4L* and *ND4* by 7 nt, and *ND5* and *ND6* (encoded on opposing stand) by 4 nt. Two ribosomal RNA genes, *12S rRNA* (953 bp) and *16S rRNA* (1679 bp) were located between *tRNA^Phe^* and *tRNA^Leu^* with separation by the *tRNA^Val^* as seen in other vertebrate mitogenomes.

To determine the taxonomic status of *M. anguillicaudatus*, we performed the phylogenetic relationship of Poyang *M. anguillicaudatus* stock with other natural populations in loach as inferred by entire mitogenome (Liang et al. 2018b). The phylogenetic tree showed that *Misgurnus* family got together for one branch, which includes Poyang *M. anguillicaudatus*, and the other loaches had their own branches (Figure 1). Also the mitochondrial genome sequence of *M. anguillicaudatus* was aligned by BLAST, compared with Cobitinae the sequence similarity could reach >95%, and the similarity to *Misgurnus* was >99%.

**Figure 1.**
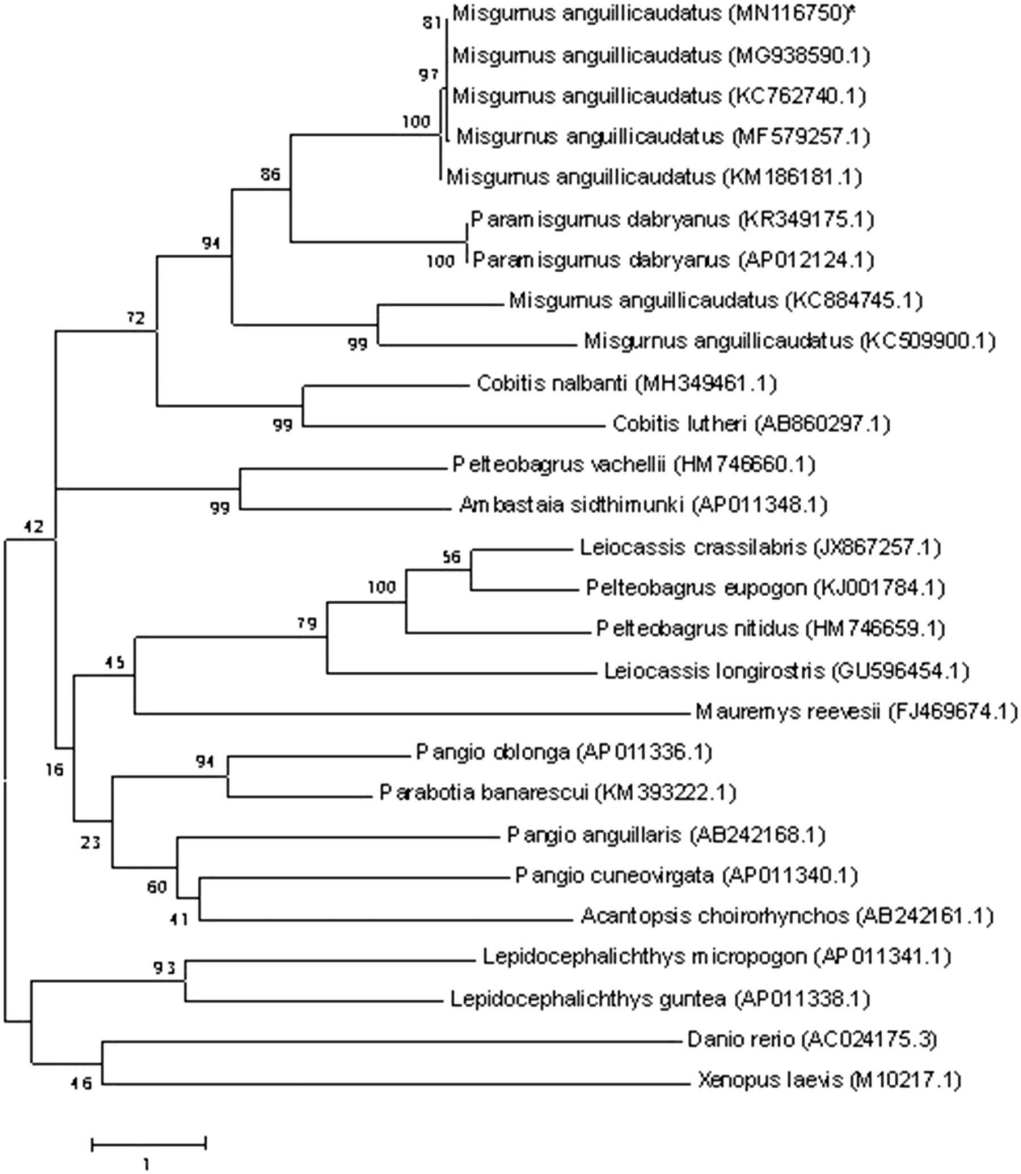
Phylogenetic relationship of Poyang *M. anguillicaudatus* stock with other loach as inferred by entire mitogenome. *The Poyang loach (accession number: MN116750) in the position of the evolutionary tree. Trees were reconstructed using MEGA 7 programme with neighbor-joining method. Numbers above branches are bootstrap values by 1000 replicates. The phylogenetic tree showed that Poyang loach to be one of *Misgurnus*, and the other loaches had their own branches.
